# Pressured to climb the social ladder: the role of popularity, social preference, and parents and friends in adolescents’ popularity-motivated aggression

**DOI:** 10.3389/fpsyg.2026.1607021

**Published:** 2026-04-14

**Authors:** Michelle F. Wright, Shruti Soudi

**Affiliations:** 1 Department of Psychology, Northern Illinois University, DeKalb, IL, United States; 2 Department of Psychology, PES University, Bengaluru, Karnataka, India

**Keywords:** bullying, cyberbullying, peer pressure, popularity, social preference, socialization

## Abstract

**Introduction:**

This study examines how perceived pressure from parents and friends to attain social status moderates the relationship between peer status (popularity, social preference) and popularity-motivated aggression (relational aggression, cyber aggression) over 1 year.

**Methods:**

A sample of 767 eighth-grade students completed self-reported measures of aggression and peer nominations of popularity and social preference.

**Results:**

Structural equation modeling showed that popularity was positively associated with both types of aggression, while social preference was negatively associated. Higher perceived pressure from parents and friends to be popular strengthened the link between popularity and aggression, whereas pressure to be socially preferred was negatively related to aggression.

**Discussion:**

These findings highlight the role of socialization agents in shaping adolescent aggression, supporting social learning theory and the dual influence model. Implications suggest the need for parental and peer interventions to mitigate status-driven aggression and encourage positive social behaviors.

## 1 Introduction

Research has linked adolescents’ attained peer status, particularly popularity and social preference, to aggressive behaviors that may function as strategies for maintaining or enhancing status within peer groups. However, adolescents’ pursuit of status does not occur in isolation. Parents and friends play an important role in shaping adolescents’ behaviors and social goals, including aggressive and bullying behaviors (Alexander et al., 2001; Allen et al., 2021; Dirikx and den Bulck, 2014; Kehoe et al., 2014; Smetana et al., 2015; Tanski et al., 2012). The present study therefore examines whether adolescents’ perceptions of pressure from parents and friends to prioritize popularity or social acceptance influence the relationship between status and aggression. In this study, the term pressure refers to adolescents’ perceptions of socialization messages and expectations from parents and friends concerning popularity and social preference. This construct captures adolescents’ sense that significant others encourage or model certain behaviors associated with being popular or well liked, rather than implying coercive or forceful influence. Consistent with previous research on the socialization of social status goals (e.g., Wiertsema et al., 2022), pressure reflects perceived encouragement to behave in ways that align with valued social outcomes. Although some studies in the literature refer to bullying, the present study focuses on aggression as the broader construct measured in the current analyses. In discussing prior research, we retain the terminology used by the original authors (e.g., bullying or aggression), whereas the term aggression is used when referring to the constructs examined in the present study.

The present study examines the moderating effect of adolescents’ perceptions of the pressure to be popular or socially preferred by parents and friends in the relationship between peer status (popularity, social preference) and popularity-motivated aggressive behaviors (relational aggression, cyber aggression), assessed 1 year later. Relational aggression is defined as behaviors intended to harm another person through damage to relationships or social status (Crick and Grotpeter, 1995); cyber aggression is defined as intentional harm (e.g., sending threatening messages, posting hurtful comments, sharing private information, excluding others online) inflicted through digital technologies, such as social media, text messages, or online platforms (Smith et al., 2008). The study controlled previous levels of popularity-motivated relational aggression and cyber aggression.

### 1.1 Popularity, social preference, and adolescent social behavior

Popularity represents a reputation-based dimension of social status, capturing how adolescents are viewed in terms of prestige, influence, and visibility within their peer group (Cillessen and Mayeux, 2004; Malamut et al., 2020). Adolescents who are perceived as highly popular tend to occupy central and influential positions within the social hierarchy, although popularity itself exists along a continuum. However, being perceived as popular does not necessarily mean an adolescent is well-liked by their peers. Research has shown that adolescents who are perceived as popular tend to engage in higher levels of aggressive and bullying behaviors compared with their peers, including but not limited to aggression toward less popular classmates (e.g., Cillessen and Mayeux, 2004; Prinstein and Cillessen, 2003; Dawes and Malamut, 2020). Other research has established a strong link between popularity and face-to-face relational bullying, which involves harming others through behaviors such as spreading rumors, manipulating friendships, and ostracizing peers (Crick and Grotpeter, 1995; Phelps et al., 2020). In contrast, some researchers suggest that popular adolescents adopt a bistrategic approach, combining both relational aggression and prosocial behavior to maintain their social status. This means they may engage in aggressive behaviors while also demonstrating prosocial actions, such as helping peers and offering emotional support when others feel down (Hawley, 2003; Prinstein, 2007; Salmivalli et al., 2021).

Unlike popularity, social preference refers to how well an individual is liked by their peer group. Social preference is characterized by high levels of social acceptance and is typically associated with behaviors that promote positive peer interactions, such as kindness and helpfulness (Chavez et al., 2022; Coie et al., 1982). Research consistently finds that social preference is negatively associated with both relational aggression and overt aggression (i.e., physical, verbal, or property damage inflicted on a peer; LaFontana and Cillessen, 2002; Prinstein and Cillessen, 2003). Additionally, social preference is positively linked to prosocial behaviors, reinforcing the idea that well-liked adolescents are more likely to engage in supportive and constructive peer interactions.

Relational aggression may be particularly relevant to peer status dynamics during adolescence. Unlike physical aggression, which tends to decline as youth progress into adolescence, relational aggression becomes a more common strategy for influencing peer relationships and reputational standing within social groups. Behaviors, such as spreading rumors, social exclusion, and reputational manipulation directly target the social connections that underlie peer status hierarchies (Crick and Grotpeter, 1995; Prinstein and Cillessen, 2003). Because popularity is inherently tied to visibility and influence within peer networks, relational aggression may function as a strategic behavior through which adolescents attempt to maintain or enhance their status among peers.

Coie et al. (1990) two-phase model of social competence provides a useful theoretical basis for understanding how popularity and social performance influence behavior. According to this model, the first phase, social acquisition, reflects the ability to understand and interpret social cues, while the second phase, social performance, reflects how effectively individuals enact behaviors to achieve desired social outcomes. Adolescents who are skilled at navigating these phases can leverage both prosocial and coercive strategies to maintain or enhance their peer status. Thus, popular adolescents may rely on relational aggression to assert dominance and control social dynamics, whereas those who are socially preferred may rely more on prosocial behaviors that foster cooperation and acceptance.

Recent work by Dawes and Malamut (2020) further supports this framework by highlighting that popularity can both protect and promote aggression depending on peer group norms. Their findings suggest that adolescents with high peer status often engage in aggression strategically, particularly relational or reputational aggression, to reinforce dominance, whereas victimization experiences may emerge for those who are less integrated or socially excluded. Integrating these perspectives emphasizes that both popularity and social preference reflect adaptive strategies within peer ecologies that shape adolescents’ behavioral choices and social adjustment. Although some research suggests that adolescents occupying high-status positions may combine aggressive and prosocial behaviors to maintain their social standing (Hawley, 2003), the present study focuses specifically on aggressive behaviors associated with status dynamics, as these behaviors align most directly with the mechanisms examined in the current model.

Theoretical frameworks of adolescent social development suggest that the social goals and behaviors adolescents pursue in offline contexts often extend to their online interactions (Subrahmanyam and Šmahel, 2011; Wright, 2015). The same motivations that drive adolescents to seek visibility, dominance, or acceptance among peers offline are frequently expressed through digital communication. For example, adolescents who are highly popular offline may use social media or online platforms to reinforce their social standing, demonstrate influence, or manage impressions among peers (Mann and Blumberg, 2022; Nesi and Prinstein, 2015). From a social learning perspective (Bandura, 1977), these behaviors can be understood as the result of observing and modeling peer norms across both offline and online contexts, where aggressive or exclusionary acts are sometimes rewarded with attention or social validation. Likewise, adolescents who are socially preferred, those high in prosociality and empathy may generalize their offline behaviors to the online environment by engaging in supportive or inclusive interactions. Thus, the social hierarchies and reputational concerns that characterize the offline peer ecology continue to shape online behaviors, influencing whether adolescents employ prosocial or aggressive strategies to maintain or enhance their social position.

Similar processes may also occur in online environments. Cyber aggression frequently involves behaviors such as spreading rumors, damaging reputations, or excluding peers through digital communication, which mirror the relational mechanisms underlying offline relational aggression. Because these behaviors target social relationships and reputational standing within peer networks, cyber aggression can function as an extension of relational aggression within digital contexts. While scholars increasingly recognize the influence of the cyber environment on adolescent development, limited research has explored how popularity and social preference relate to aggressive and prosocial behaviors in online settings (Smahel et al., 2014); Sobkin and Fedotova, 2021). Adolescents are among the highest consumers of electronic technology and often leverage digital platforms to enhance their peer status (Haddock et al., 2022; Trekels et al., 2024; Wright, 2015).

Existing research suggests that popularity is positively associated with cyber aggression. For example, popular adolescents are more likely to engage in online behaviors that harm their peers, such as cyberbullying (Lapierre and Dane, 2020; Schoffstall and Cohen, 2011). However, there is little research on how social preference relates to cyberbullying. The limited evidence available suggests that social preference follows similar patterns online as it does offline. In one study, Wright (2014) found that social preference was positively associated with cyber prosocial behavior but negatively associated with cyber aggression. Furthermore, Wright’s findings indicated that popularity was related to cyber aggression. Despite these insights, there remains a gap in understanding the role of socialization agents (e.g., parents, friends) in shaping adolescents’ attitudes toward peer status and their subsequent behaviors in both face-to-face and cyber settings. Further research is needed to explore how external influences contribute to the development of popularity-driven and social preference-driven behaviors in offline and online environments.

### 1.2 The influence of socialization agents on popularity and social preference

Parents are primary socialization agents in their children’s lives, shaping their attitudes and behaviors through direct and indirect influence (Kehoe et al., 2014; Smetana et al., 2015). Children often adopt behaviors that reflect their parents’ values, yet few studies have examined how parents specifically socialize their children’s pursuit of popularity and social preference. Most research on parental influence in this domain has focused on identifying characteristics that parents believe contribute to their child’s popularity, such as friendliness, academic success, and physical attractiveness (Ridao et al., 2021; Tatar, 1995). However, this research does not distinguish between factors influencing popularity versus social preference, nor does it explore whether adolescents’ perceptions of their parents’ attitudes about popularity affect their aggressive behaviors. To date, no studies have examined whether adolescents perceive their parents as actively encouraging or pressuring them to attain a particular social status within their peer group.

Peers and friends also play a critical role in shaping adolescents’ social status. Much of the existing research has focused on peer pressure, particularly its relationship with problem behaviors, such as alcohol consumption. Studies have shown that adolescents who experience greater peer pressure and have a strong desire for popularity are more likely to engage in risky behaviors (McKay and Cole, 2012; Schaefer et al., 2013). Additionally, research indicates that adolescents often emulate the behaviors of their closest friends. For instance, adolescents whose best friends smoked cigarettes were more likely to start smoking themselves (Alexander et al., 2001). Similarly, affiliation with peers who engage in risky sexual behaviors has been linked to adolescents’ own sexual behaviors and increased need for popularity (Allen et al., 2006); in addition, Andrews et al. (2020) found that adolescents’ peers impacted their social distancing behaviors during the COVID-19 pandemic. Peer conformity and pressure have also been found to influence adolescents’ sexual attitudes, as well as their engagement in behaviors such as sexting and viewing pornography on mobile devices (Santer et al., 2000; Vanden et al., 2014). Despite this growing body of research, little attention has been given to whether adolescents’ perceptions of their friends’ attitudes about popularity and social preference influence their aggressive behaviors.

It is also unclear how attained peer status, such as popularity and social preference, relates to adolescents’ perceptions of pressure from parents and friends to be popular or socially preferred. However, it might be expected that such pressure could influence the association between attained status and popularity-motivated face-to-face relational aggression and cyber aggression.

These patterns can be understood through social learning theory (Bandura, 1977), which suggests that individuals acquire behaviors through observation and reinforcement from influential social agents. Adolescents are particularly susceptible to modeling behaviors that are valued within their social environments, including aggressive strategies used to enhance or maintain peer status. When parents and friends explicitly or implicitly communicate the importance of social status, adolescents may internalize these messages and engage in relational aggression or cyber aggression as a means of securing or elevating their social position. Through reinforcement mechanisms such as peer approval or increased social visibility, these aggressive behaviors may become ingrained as strategic tools for navigating peer hierarchies.

In addition, the dual influence model (Prinstein and Dodge, 2008) provides further insight into how socialization agents shape adolescents’ aggression in the context of popularity. This model highlights both direct and indirect influences on adolescent behavior. Directly, adolescents may receive explicit encouragement from parents or peers to prioritize popularity, reinforcing behaviors that have been previously associated with high-status individuals (Vaillancourt and Hymel, 2006). Societal and cultural norms that emphasize social dominance and status-driven behaviors contribute to adolescents’ perceptions of acceptable ways to navigate peer interactions (Dumas et al., 2019). The pressures to conform to these social expectations may drive adolescents to use relational aggression and cyber aggression as mechanisms for achieving or maintaining their status.

Furthermore, popularity-driven aggression in digital contexts may also be interpreted through the lens of the online disinhibition effect (Suler, 2004). This phenomenon suggests that the online environment fosters reduced self-regulation and accountability, enabling adolescents to engage in more aggressive behaviors than they might in face-to-face interactions. Given that social media platforms amplify visibility and peer approval mechanisms (e.g., likes, shares, and comments), adolescents who perceive pressure to attain or sustain popularity and social preference may be more likely to exploit these digital spaces for aggressive and prosocial social maneuvering (Mann and Blumberg, 2022; Nesi and Prinstein, 2015).

### 1.3 Present study

**Figure 1:**
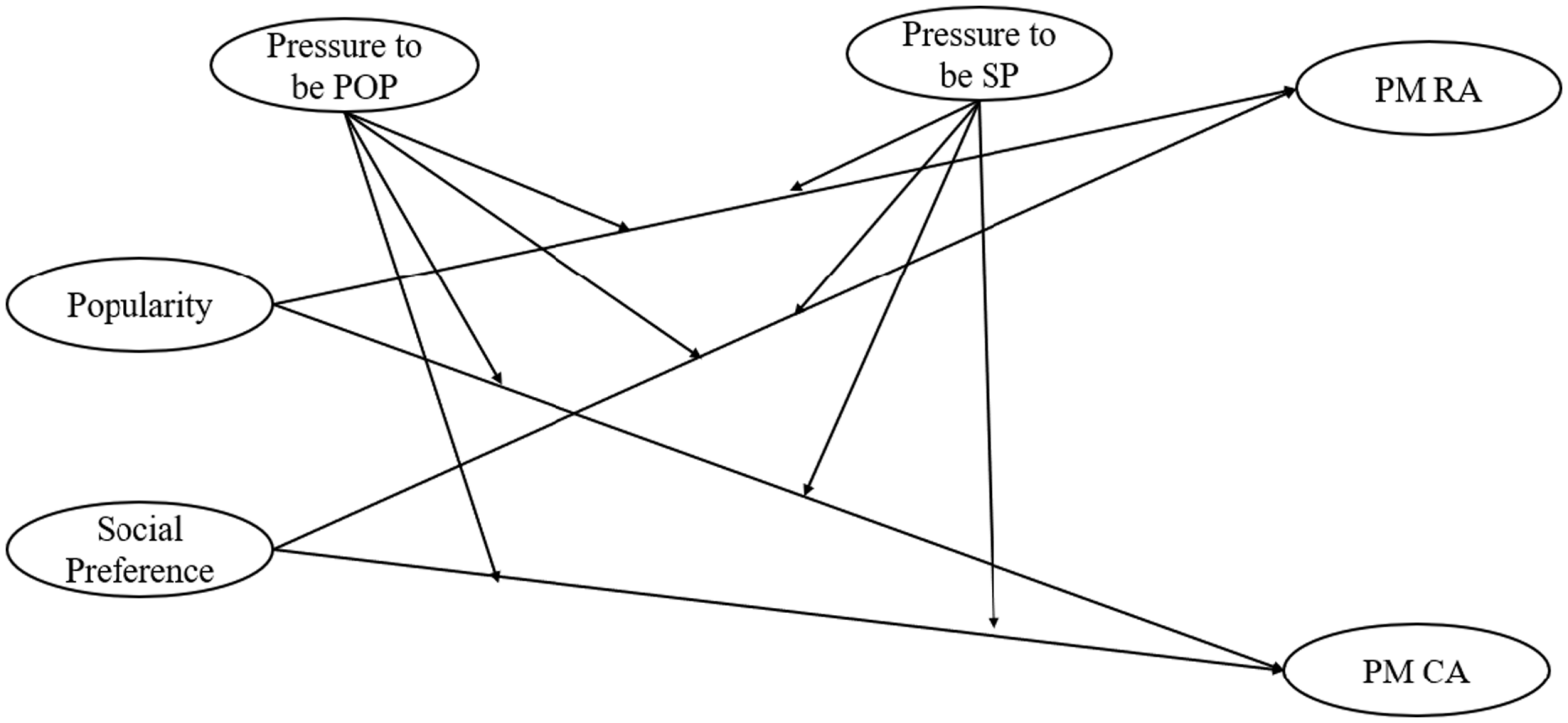
Proposed structural regression model. POP, popularity; SP, social preference; PM, popularity-motivated; RA, relational aggression; CA, cyber aggression.

The present study examined whether perceived pressure from parents and friends shapes the longitudinal association between adolescents’ attained popularity and social preference and their engagement in popularity-motivated relational bullying and cyber aggression 1 year later. By considering both offline and online forms of aggression, this study aimed to better understand how peer status dynamics unfold across social contexts during adolescence. Drawing on the empirical and theoretical foundations reviewed above, we expected that attained popularity would be positively associated with subsequent relational bullying and cyber aggression, whereas social preference would be negatively associated with these behaviors. We further anticipated that perceived pressure from parents and friends to prioritize popularity would intensify the association between attained popularity and aggression over time. In contrast, perceived pressure to prioritize being socially preferred was expected to buffer against status-driven aggression. Figure 1 provides a visual depiction of the relationships examined in this study.

## 2 Methods

### 2.1 Participants

This study included 767 eighth grade students (50.8% female) from seven middle schools (grades 6–8) in the Midwestern United States. Participants ranged in age from 12 to 15 years, with a mean age of 12.89. Most participants identified as White (71.1%), followed by Latino/a (14.4%), Black/African American (7.1%), Asian (6%), American Indian or Alaskan Native (1.2%), and Native Hawaiian or Other Pacific Islander (0.2%). The students primarily came from middle-class neighborhoods. A convenience sample was utilized, and there were no inclusion or exclusion criteria for this study, other than students attending a participating middle school, being in the seventh grade at the beginning of the study and having parental permission.

### 2.2 Procedures

This study received approval from the university’s Institutional Review Board. A list of over 200 middle schools in the suburbs of a large Midwestern city was compiled. From this list, 10 schools were randomly selected, and an email was sent to their principals outlining the study’s purpose, participation requirements, and the significance of student involvement. Seven principals expressed interest in participating, while three declined due to commitments to other research projects. Two of the seven schools required district-level approval, which was successfully obtained, while the remaining five did not require such approval. School principals, along with seventh and eighth teachers were then briefed on the study’s objectives and student participation expectations. Classroom announcements were made, and students received letters and parental consent forms to take home. A total of 961 letters and consent forms were distributed; 857 were returned with consent, 40 were returned without consent, and 64 were not returned at the first time point (Time 1; Spring 2023), during the seventh grade.

At Time 1, data collection took place over 6 weeks at the participating schools. Before completing the measures, students provided assent, with all participants agreeing to take part. Ten students were absent on the initial day of data collection, so a make-up session was arranged at five schools to include these students. On the day of data collection, participants completed questionnaires covering demographic information (e.g., age, gender, ethnicity), self-reported popularity-motivated face-to-face aggression and cyber aggression, perceived pressure to be popular from parents and friends (i.e., Popularity and Social Preference Pressure Conformity Measure), and peer nominations for attained popularity and social preference.

One year later (Time 2; Spring 2024), during the eighth grade, a reminder letter was sent home to parents/guardians. The letter reminded parents/guardians about their child’s participation in the study the year prior and indicating to them that their child is invited to a follow-up study. If parents/guardians wanted to opt their child out of the study, they were asked to write the first and last name of the child and then return it to their child’s school. Fifteen letters were returned to school, expressing the desire to unenroll their child from the study. Sixty-three adolescents were no longer students at the participating school, and thus, they were dropped from participating, due to inability to follow them up. The remaining 12 students were absent or unavailable on the 2 days of data collection, and therefore, they were not enrolled further in the study. Thus, the final sample at Time 2 was 767 adolescents. For Time 2, adolescents completed assent documents once more, all agreed to participate, and questionnaires on their self-reported popularity-motivated face-to-face aggression and cyber aggression.

### 2.3 Measures

#### 2.3.1 Self-reported popularity-motivated face-to-face relational aggression

This 9-item measure assessed how frequently students engaged in relational aggression (e.g., “Spread untrue and bad rumors about a peer,” “Gossip about a peer,” “Ignore a peer,” “Insult a peer”) to become popular in the offline environment. Responses were rated on a 5-point Likert scale (1 = *not at all*, 5 = *all of the time*) (Wright et al., 2014). The internal consistency was high, with Cronbach’s alpha of 0.86 for relational aggression at Time 1; Cronbach’s alpha was 0.83 for relational aggression at Time 2.

#### 2.3.2 Self-reported popularity-motivated cyber aggression

A similar measure assessed how often adolescents engaged in cyber aggression (9 items; e.g., “Spread untrue and bad rumors about a peer online or via text,” “Gossip about a peer online to damage their reputation,” “Ignore a peer online,” “Insult a peer via text or online messages”) to become popular in the online environment (Wright, 2014). Responses were rated on the same 5-point Likert scale, ranging from 1 (*never*) to 5 (*all the time*). The reliability was strong, with Cronbach’s alphas of 0.89 for both time points.

#### 2.3.3 Popularity and social preference pressure conformity

Before answering questions, participants read descriptions of social preference (e.g., being likeable, kind, helpful, and concerned for others) and popularity (e.g., being socially central and frequently imitated by peers; Wright, 2015). They then reported the level of pressure they felt from parents and friends to be socially preferred or perceived as popular. Each question followed the format: “____ encourages me to be…in order to be popular.” The blank was replaced with either “parents,” or “friend,” and the phrase “be popular” was substituted with “be socially preferred” or “be perceived as popular.” Sample items included: “My friends encourage me to be nice to my peers so that I can be popular” for social preference and “My friends encourage me to wear certain clothes so that I can be perceived as popular” for popularity. Each version of the questionnaire (parents, friends) contained five items: two assessing social preference pressure and three assessing popularity pressure. Responses were rated on a 5-point scale (1 = *totally untrue*, 5 = *totally agree*). The measure demonstrated good reliability, with Cronbach’s alphas ranging from 0.81 for popularity pressure from parents and 0.88 for popularity pressure from friends.

The pressure measures capture adolescents’ perceptions that parents and peers communicate expectations regarding how to attain popularity or social acceptance. The behavioral examples included in the items (e.g., being nice, wearing certain clothes) represent behaviors adolescents commonly associate with achieving these social goals. Importantly, these items do not assess encouragement of aggression; rather, they capture perceived socialization messages regarding status attainment, which may indirectly shape adolescents’ behavioral strategies.

#### 2.3.4 Peer nominations of social preference and popularity

Participants nominated peers based on four descriptions: “Peers they liked the most” and “peers they liked the least” for social preference (Coie et al., 1982), and “Peers they considered popular” and “peers they considered unpopular” for popularity (Cillessen and Mayeux, 2004). All participants received a piece of paper with a list of all students in their grade and an associated ID code. Instead of recording names on this questionnaire, participants recorded the ID code, and they were allowed to nominate as many peers as they believed fit the description, if the peer is in their grade and at their school. Nomination counts were standardized to account for school size. Social preference scores were calculated by subtracting the standardized “like least” nominations from “like most” nominations. Similarly, popularity scores were derived by subtracting “unpopular” nominations from “popular” nominations. This measure was administered at Time 1 only.

### 2.4 Analytic plan

Before testing hypotheses using a structural equation modeling, the measurement model was examined through confirmatory factor analysis (CFA) using *Mplus 6.12*. The CFA indicated a good model fit: *χ*^2^ = 930.99, d*f* = 736, *p* < 0.05, CFI = 0.99, TLI = 0.99, RMSEA = 0.04, SRMR = 0.03. All standardized factor loadings were significant (*p* < 0.001) with adequate magnitudes, confirming that the items reliably measured the intended constructs. These items were then used as indicators for latent variables in the model. To further evaluate the adequacy of the measurement model, composite reliability (CR) and average variance extracted (AVE) were calculated for each latent construct. All constructs demonstrated acceptable composite reliability (CRs ranged from 0.82 to 0.91), indicating strong internal consistency across indicators. AVE values ranged from 0.54 to 0.72, exceeding the recommended threshold of 0.50 and supporting convergent validity. These findings suggest that the latent constructs captured a substantial proportion of shared variance among their respective indicators, providing additional confidence in the distinctiveness and stability of the measurement model.

A structural equation model was used to test the study’s hypotheses. The model included paths from Time 1 attained social preference and popularity to Time 2 self-reported popularity-motivated face-to-face and cyber aggression, as well as from popularity pressure from parents and friends to Time 2 self-reported popularity-motivated face-to-face and cyber aggression. To control for Time 1 self-reported popularity motivated face-to-face and cyber aggression, these variables were allowed to predict their Time 2 receptive behaviors. Additionally, we specified interactions between attained social preference and popularity pressure from parents, social preference and popularity pressure from friends, popularity and popularity pressure from parents, and popularity and popularity pressure from friends. Each interaction term was included in the structural model to examine whether the associations between adolescents’ attained social status (popularity or social preference) and popularity-motivated aggression varied as a function of perceived pressure from parents and friends. Significant interactions were probed using simple slopes analyses at low (−1 SD), mean, and high (+1 SD) levels of the moderator to clarify the direction and magnitude of the effects.

## 3 Results

Before conducting the main analyses, descriptive statistics and assumptions were examined. Skewness and kurtosis values for all continuous variables were within acceptable ranges (±2), indicating no substantial deviations from normality. Correlation analyses indicated that popularity was positively associated with social preference and popularity pressure from parents and friends to be popular, but unrelated to popularity pressure from parents and friends to be social preferred (see Table 1). Social preference was unrelated to popularity pressure from parents and friends to be popular, but it was positively associated with popularity pressure from parents and friends to be socially preferred. The findings also revealed that adolescents who engaged in both Time 1 and Time 2 popularity-motivated face-to-face relational and cyber aggression were more likely to have higher perceived popularity. Conversely, adolescents who engaged in Time 1 and Time 2 popularity-motivated face-to-face relational and cyber aggression tended to report lower social preference. Popularity pressure from parents and friends to be popular were associated positively with each other, as well as Time 1 and Time 2 popularity-motivated relational and cyber aggression. Popularity pressure from parents and friends to be socially preferred were unrelated to each other, but both were negatively associated with Time 1 and Time 2 popularity-motivated relational and cyber aggression. Additionally, adolescents who engaged in face-to-face aggression at Time 1 and Time 2 were also more likely to perpetrate popularity-motivated cyber aggression at Time 1 and Time 2.

**Table 1:** Correlation among variables.

	1	2	3	4	5	6	7	8	9	10
1. POP	—									
2. SP	0.22*	—								
3. Pressure to be POP from parents	0.30***	0.12	—							
4. Pressure to be POP from friends	0.40***	0.16	0.19*	—						
5. Pressure to be SP from parents	0.10	0.26**	0.06	0.05	—					
6. Pressure to be SP from friends	0.08	0.30***	0.07	0.04	0.14	—				
7. Time 1 PM RA	0.36***	−0.29**	0.33***	0.37***	−0.26**	−0.20*	—			
8. Time 1 PM CA	0.31***	−0.22*	0.29**	0.35***	−0.19*	−0.20*	0.43***	—		
9. Time 2 PM RA	0.35***	−0.29**	0.30***	0.34***	−27**	−0.23*	0.33***	0.34***	—	
10. Time 2 PM CA	0.31***	−0.25*	0.30***	0.33***	−0.21*	−0.19*	0.36***	0.36***	0.44***	—

POP, popularity; SP, social preference; PM, popularity-motivated; RA, relational aggression; CA, cyber aggression.

*p < 0.05; **p < 0.01; ***p < 0.001.

### 3.1 Main effects

The structural model demonstrated an acceptable fit to the data, *χ*^2^ (808) = 1131.35, *p* < 0.001, CFI = 0.99, TLI = 0.98, RMSEA = 0.05, SRMR = 0.05 (see Table 2). Multicollinearity was evaluated using variance inflation factor values, all of which were below the recommended cutoff of 5, suggesting no issues with collinearity among predictors. These preliminary checks supported the appropriateness of the model estimation method. The model accounted for 39% of the variance in Time 2 popularity-motivated relational bullying (*R*^2^ = 0.39) and 27% of the variance in Time 2 popularity-motivated cyber aggression (*R*^2^ = 0.27). When controlling Time 1 popularity-motivated relational and cyber aggression, popularity was related positively to Time 2 popularity-motivated relational and cyber aggression. On the other hand, social preference was associated negatively with both types of popularity-motivated behaviors. The pressure to be popular from parents and friends were both associated positively with both behaviors, while the pressure to be socially preferred by parents and friends were related negatively with popularity-motivated face-to-face relational and cyber aggression.

**Table 2:** Structural equation model.

	Time 2 PM RA		Time 2 PM CA	
	*β*	SE	*β*	SE
Time 1 PM RA	0.20***	0.07	—	—
Time 1 PM CA	—	—	0.20***	0.08
POP	0.26***	0.10	0.21***	0.07
SP	−0.16**	0.06	−0.13**	0.04
Pressure to be POP from parents	0.18**	0.08	0.18**	0.08
Pressure to be POP from friends	0.23***	0.09	0.25***	0.10
Pressure to be SP from parents	−0.10*	0.03	−0.09*	0.03
Pressure to be SP from friends	−0.10*	0.03	−0.08*	0.02
POP × pressure to be POP from parents	0.11*	0.04	0.10*	0.03
SP × pressure to be POP from parents	−0.03	0.01	−0.02	0.01
POP × pressure to be POP from friends	0.16**	0.06	0.15**	0.05
SP × pressure to be POP from friends	−0.04	0.02	−0.03	0.01
POP × pressure to be SP from parents	0.01	0.01	0.02	0.01
SP × pressure to be SP from parents	0.04	0.02	0.03	0.01
POP × pressure to be SP from friends	0.01	0.01	0.01	0.01
SP × Pressure to be SP from friends	−0.02	0.01	0.03	0.01

POP, popularity; SP, social preference; PM, popularity-motivated; RA, relational aggression; CA, cyber aggression.

*p < 0.05; **p < 0.01; ***p < 0.001.

### 3.2 Interaction effects

The interactions between popularity and the pressure to be popularity from parents and friends when predicting Time 2 popularity-motivated relational and cyber aggression were significant. Probing the interaction further indicated that the link between attained popularity and subsequent aggression was amplified at higher levels of perceived pressure to prioritize popularity. At high levels of parental pressure, attained popularity was positively associated with relational bullying [*B* = 0.07, SE = 0.02, *p* = 0.021, 95% CI (0.03, 0.11)] and cyber aggression [*B* = 0.05, SE = 0.02, *p* = 0.028, 95% CI (0.01, 0.09)]. A similar pattern emerged for peer pressure, such that popularity more strongly predicted relational bullying [*B* = 0.10, SE = 0.03, *p* = 0.002, 95% CI (0.04, 0.16)] and cyber aggression [*B* = 0.09, SE = 0.03, *p* = 0.013, 95% CI (0.03, 0.15)] when perceived peer pressure to be popular was high. Lower and middle levels of this type of popularity pressure were not significant, nor were the interactions for popularity pressure from parents and friends to be socially preferred.

## 4 Discussion

The present study examined adolescents’ attained status (popularity and social preference), their perceptions of pressure from parents and friends to become more popular or socially preferred, and how these factors interact to shape engagement in popularity-motivated relational bullying and cyber aggression 1 year later. Importantly, the behaviors examined here reflect aggression enacted with the goal of increasing or maintaining popularity, rather than aggression in a general sense. Thus, the findings should be interpreted within this specific motivational framework.

The findings regarding adolescents’ attained peer status highlight the distinct roles of popularity and social preference in shaping aggressive behavior. Consistent with prior literature (e.g., Cillessen and Mayeux, 2004; Coie et al., 1982; Crick and Grotpeter, 1995; LaFontana and Cillessen, 2002; Prinstein, 2007; Prinstein and Cillessen, 2003), attained popularity was positively associated with popularity-motivated relational bullying and cyber aggression 1 year later, even after accounting for prior behavior. Adolescents who occupied positions of higher visibility and influence were more likely to engage in aggression aimed at sustaining or enhancing their status. This pattern aligns with theoretical and empirical work suggesting that dominance-based status can, at times, be maintained through strategic social behaviors (Vaillancourt and Hymel, 2006; Malamut et al., 2020). In other words, for some adolescents, aggression may function as a tool within the broader pursuit of popularity.

In contrast, social preference demonstrated a different pattern. Higher levels of social preference were associated with lower engagement in popularity-motivated relational bullying and cyber aggression. This distinction is meaningful. Popularity and social preference are often discussed together, yet they reflect fundamentally different dimensions of peer status (Cillessen and Mayeux, 2004; LaFontana and Cillessen, 2002). Popularity is rooted in visibility, influence, and perceived power, whereas social preference reflects affective acceptance, being genuinely liked by peers (Coie et al., 1982). Adolescents who are broadly liked may have less need to rely on dominance-oriented strategies to maintain their position. This interpretation is consistent with research showing that adolescents involved in bullying roles often occupy complex social positions in which high popularity may coexist with lower social preference (Pouwels et al., 2018). Similarly, research examining bullying roles across classrooms has found that perceived popularity and social preference operate differently depending on peer norms and social contexts (Romera et al., 2019). Together, these findings highlight that dominance-based status and affect-based acceptance represent separate, and at times divergent, dimensions of adolescent social hierarchies. Such findings reinforce that visibility and influence do not necessarily translate into genuine peer acceptance. The affective foundation of social preference may therefore serve as a protective factor against engaging in aggression aimed at increasing popularity.

These status-related dynamics were evident not only in face-to-face contexts but also in digital environments. The association between popularity and popularity-motivated cyber aggression suggests that online spaces may represent additional arenas in which status processes unfold (Wright, 2014). Given the public and amplified nature of social media, where visibility, feedback, and peer reactions are immediate and measurable, adolescents seeking to maintain or elevate their popularity may perceive these platforms as strategic spaces for social maneuvering (Nesi and Prinstein, 2015). Understanding adolescent behavior therefore requires attending to both offline and online interactions, as status dynamics extend across multiple social contexts.

Perceived socialization pressures from parents and friends further clarified how these status processes operate. Adolescents who perceived greater parental pressure to be popular were more likely to engage in popularity-motivated relational bullying and cyber aggression. In contrast, perceived parental pressure to be socially preferred was negatively associated with these same behaviors. These findings are consistent with research highlighting parents as central socialization agents who shape adolescents’ values and behavioral expectations (Allen et al., 2021; Kehoe et al., 2014; Smetana et al., 2015). When popularity is emphasized within the family context, adolescents may perceive dominance-oriented strategies as acceptable or even advantageous for achieving social success. Conversely, when parents emphasize being well liked and maintaining positive relationships with peers, adolescents may be less inclined to adopt aggressive strategies aimed at enhancing popularity.

A similar pattern emerged for peer influence. Perceived pressure from friends to prioritize popularity was associated with greater engagement in popularity-motivated aggression, whereas pressure to be socially preferred was associated with lower engagement. Given the well-documented role of peers in shaping adolescent norms and behaviors (McKay and Cole, 2012; Schaefer et al., 2013; Santer et al., 2000), these findings suggest that adolescents’ behavioral strategies may be shaped not only by their own status positions but also by the expectations embedded within their immediate peer networks. Adolescents are highly attuned to peer norms and approval, and perceived expectations from friends may reinforce certain behaviors as effective strategies for maintaining or enhancing social standing.

The moderation findings provide additional insight into how status and socialization pressures interact to shape adolescents’ behavior. Perceived pressure to become popular amplified the association between attained popularity and popularity-motivated aggression. Adolescents who were already high in popularity and who also perceived stronger social pressure to maintain that status were particularly likely to engage in aggression aimed at reinforcing their position. This finding highlights that status processes are not static; rather, they are shaped by adolescents’ interpretations of social expectations. Popularity alone does not inevitably lead to aggression. Instead, its behavioral expression appears contingent on the surrounding social messages adolescents perceive. This pattern is consistent with social learning theory (Bandura, 1977), which emphasizes reinforcement from influential agents, as well as the dual influence model (Prinstein and Dodge, 2008), which highlights the interplay of direct and indirect socialization processes.

At a broader level, the present findings contribute to a more nuanced understanding of adolescent peer status. By examining both popularity and social preference within the same model, this study demonstrates that these constructs function differently in relation to aggression enacted for popularity enhancement. Popularity appears linked to strategic, dominance-based behaviors, particularly when reinforced by perceived social pressure. Social preference, in contrast, appears grounded in affective acceptance and may buffer against the use of aggression as a status-seeking strategy. Had the focus been limited solely to popularity, this important distinction may have remained obscured.

At the same time, because the present study examined aggression specifically motivated by popularity, it remains an open question whether similar patterns would emerge for general aggression or aggression enacted in pursuit of other social goals, including maintaining social preference. Future research should continue to disentangle how different social motivations shape adolescents’ behaviors across both offline and digital contexts.

### 4.1 Limitations and future directions

This study provides important insights into attained peer status and adolescents’ perceptions of social pressures from parents and friends in relation to their popularity-motivated aggression. One limitation concerns the reliance on adolescent self-report measures, which may be influenced by social desirability or recall bias. Future studies should include multiple informants, such as parents, teachers, or peers, to capture a more comprehensive view of adolescents’ social behaviors. In addition, the relatively short time frame of the study restricts the ability to identify causal relationships between perceived social pressure and aggression, emphasizing the need for longer longitudinal designs. Finally, the homogeneity of the sample limits the generalizability of findings; future research should aim to recruit participants from more diverse cultural, ethnic, and socioeconomic backgrounds to strengthen external validity. Future research should employ mixed-methods approaches that integrate quantitative surveys with qualitative interviews or focus groups to better understand how adolescents interpret and negotiate social pressure from parents and peers. Cross-cultural studies would also be valuable to determine whether these dynamics vary across different cultural contexts and social norms regarding popularity and conformity. Moreover, examining potential protective factors, such as empathy, emotional regulation, or parental warmth, could clarify why some adolescents resist pressure to engage in popularity-driven aggression. Longitudinal studies extending across multiple developmental periods could further illuminate how perceived social pressure evolves from early to late adolescence and influence long-term social outcomes, especially considering that attained popularity and social preference were measured at time one only. Researchers interested in such a longitudinal study might also consider trying to determine whether there are differences in the manifestation of pressure from parents and friends when it comes to attained social preference and popularity. Taken together, addressing these limitations will advance understanding of how social and familial pressures shape adolescents’ pursuit of popularity and associated behaviors. A more nuanced understanding of these processes could inform the design of prevention and intervention programs in schools and families that target social motivation, peer dynamics, and prosocial goal development, ultimately helping reduce aggression linked to popularity striving.

### 4.2 Practical implications

The present findings carry meaningful implications for parents, educators, and practitioners who seek to reduce relational and cyber forms of aggression among adolescents. First, prevention and intervention efforts may benefit from addressing not only aggressive behaviors themselves, but also the social motivations that underlie them. For some adolescents, aggression appears to function as a strategy for sustaining visibility and status. Helping youth reflect on the distinction between being popular and being genuinely well-liked may reduce the perceived need to engage in status-driven tactics.

Second, parents and caregivers play an influential role in shaping adolescents’ interpretations of peer status. Even subtle messages that prioritize popularity may be internalized in ways that reinforce competitive or dominance-oriented strategies. Encouraging conversations that emphasize empathy, cooperation, and mutual respect may help buffer adolescents from engaging in aggression as a means of social advancement.

Finally, schools and youth programs may consider integrating social–emotional learning and digital citizenship initiatives that explicitly address how peer dynamics unfold both offline and online. Given the strong continuity observed between face-to-face and digital status processes, interventions that attend to both environments are likely to be more effective. Supporting adolescents in developing prosocial ways of achieving recognition and belonging may ultimately foster peer cultures that value inclusion over competition.

## 5 Conclusion

This study advances our understanding of how adolescents navigate peer hierarchies by examining how attained popularity and social preference, along with perceived pressure from parents and friends, shape engagement in popularity-motivated relational bullying and cyber aggression. The findings suggest that popularity and social preference operate as distinct dimensions of social status, each associated with different behavioral pathways. Adolescents who had attained higher popularity were more likely to engage in aggression enacted to maintain or enhance that status, particularly when they perceived social pressure to prioritize popularity. In contrast, social preference was negatively associated with popularity-motivated aggression, suggesting that being genuinely liked may reduce reliance on dominance-based strategies. Together, these findings highlight that adolescents’ behaviors are shaped not only by where they stand within peer hierarchies, but also by how they interpret the expectations communicated by the important people in their lives.

## Data Availability

The raw data supporting the conclusions of this article will be made available by the authors, without undue reservation.
